# Exploring potential benefits of social participation for work and life outcomes: Drawing on the Health and Retirement Study

**DOI:** 10.21203/rs.3.rs-7022532/v1

**Published:** 2025-07-10

**Authors:** Christina Christodoulou

**Keywords:** Social participation, wellbeing, work ability, life satisfaction, aging workforce, occupational health

## Abstract

Considering the trend of an aging population, it is important that researchers and practitioners aim to optimize and maintain individuals’ wellbeing over the course of their careers and life. Preventative medicine and occupational health research have determined that participation in one’s social environment tends to yield positive outcomes in the realm of cognitive functioning; however, in order to make these findings useful for employees and, by extension, their organizations, determining the nature of such relationships should benefit health and wellbeing research. Therefore, the current research draws on national data from 2018 and 2020 waves of the Health and Retirement Study (HRS) to investigate potential drivers of the relationship between social participation and work and life outcomes. Participants (2018 N = 1,933; 2020 N = 1,427) included working individuals aged over 50 years. Findings suggest that sense of control, purpose in life and optimism may play an important role in mediating the influence of social participation on both work ability and life satisfaction. Importantly, results replicated between 2018 and 2020 waves of data. Theoretical and practical implications that have relevance to a broad audience, including researchers, organizations and employees, are offered.

## Introduction

It is well established that there is a positive relationship between social participation, which describes the frequency of engagement in one’s social environment (Health and Retirement Study), and positive outcomes, particularly in the context of the wellbeing and cognitive patterns of aging workers (Jiang et al., 2020). Specifically, research indicates that engagement in one’s social environment can enhance affect, cognition, resilience, creativity and health (Wang & Boros, 2021; Jiang et al., 2020; Cadiz et al., 2018). Despite the proliferation of research on the importance of work-life balance, the specific benefits surrounding the construct of social participation remain unclear (Jiang et al., 2020). Importantly, a loss of cognitive functioning and independence in activities, which often coincides with unhealthy aging, has been referred to as a public health concern (Fischer et al., 2017). According to Fisher et al. (2017), “It is critical to identify risk and protective factors related to cognitive health and the maintenance of cognitive functioning because they contribute to successful aging” (p. 315). Hence, resources surrounding the continuous maintenance and sharpening of cognitive functioning are paramount to achieving sustainable work performance and optimal life outcomes.

While social activity has been noted as an important antecedent of cognitive functioning (Fischer et al., 2017), health and psychological wellbeing (Zacher, 2015), current models of successful aging at work (e.g. Zacher, 2015; Kooij et al., 2020) do not yet include individual lifestyle habits such as social participation—suggesting a need for understanding the explanatory mechanisms that support relationships between social participation and work and life outcomes. Therefore, the current research expands on prior work by identifying pathways for the relationships between social participation and work and life outcomes. Specifically, the current work evaluates having a sense of control, purpose in life, and optimism as mediators of the influence of social participation.

The Health and Retirement Study has been a rich source of information for understanding the psychosocial and work environmental factors that influence occupational health and wellbeing (Zahn et al., 2009; Fisher et al., 2014). Using this data, three primary contributions are made: First, the growing relevance of personal resources and the importance of social participation as a personal resource are emphasized. Second, a potential roadmap underscoring the nature of the work and personal life benefits of social participation is uncovered. Finally, a variety of theoretical and practical recommendations that are designed to aid both employees and organizations are offered. Importantly, all contributions are derived from a replication of results between 2018 and 2020 waves of data.

The current study’s rationale is guided by the environmental complexity hypothesis, which posits that diverse environmental stimuli fosters the maintenance and improvement of cognitive functioning (Hultsch et al., 1999). Social engagement in a variety of settings can buffer the consequences of stress while boosting mood, self-esteem and health (Wang & Boros, 2021). Individuals who frequently participate in their social environments are likely to experience a host of benefits, particularly in the context of longevity and wellbeing (Wang & Boros, 2021; Jiang et al., 2020; Cadiz et al., 2018). Hence, work ability (i.e., a construct that captures workplace longevity) and life satisfaction (i.e., a construct that captures one’s fulfillment in life) should increase as a function of social participation. Therefore, the current work specifically aims to provide empirical evidence regarding the explanatory mechanism through which social participation impacts work ability and life satisfaction—contributing to a better understanding of successful aging in and outside of work.

### Supporting theoretical frameworks

#### The Jobs Demands-Resources Model (JD-R)

The job demands-resources model (JD-R; Bakker & Demerouti, 2007) categorizes every aspect of one’s work as either a demand or a resource. Employees are inevitably exposed to cognitive, physical, social and emotional demands throughout each workday. To meet demands, employees are required to expend resources (Hynes & Koc, 2024), which are defined as features that aid employees’ work engagement, goal achievement, demand management and stress recovery (Brady et al., 2020; Kondrysova et al., 2021). Fittingly, individuals’ responses to demands are, in large part, controlled by their momentary availability of resources (Firoozabadi et al., 2018). Considering that much of one’s waking time is spent at work (Kelly et al., 2020), their wellbeing may, at least in large part, vary as a function of their work experiences and ability to recover from work. Hence, resources aimed at stress reduction in a work context are likely to facilitate similar benefits in a general life context through positive spillover (Sonnentag & Fritz, 2015).

Whereas the set of resources categorized as job resources are offered by the organization and include vacation time, incentives, social support and job autonomy, the dimension of personalresources describes features that exist under one’s own relative control (e.g. health, mindset, resilience) (Bakker & Demerouti, 2007; Park et al., 2011). Interestingly, while engagement in one’s social environment has been connected to mental and physical health (Carlson, 2021)—features described as personal resources, social participation has not yet been categorized as a personal resource (McGonagle et al., 2015). In addition, personal resources in general are understudied in the literature and of growing relevance to the modern worker (Cavallari et al., 2024; Dugan et al., 2021). Therefore, a deeper understanding of the nature and benefits of personal resources is required to modernize stress and wellbeing fields and best equip practitioners to improve worker wellbeing in the forms of life satisfaction and work ability.

#### The Motivational Theory of Lifespan Development

The motivational theory of lifespan development (MTLD; Heckhausen et al., 2010) proposes that people strive to maintain a sense of control over their lives, and goal-directed behaviors are most beneficial when they impact multiple domains (Heckhausen et al., 2019). According to MTLD, when people engage in activities designed to increase their sense of control (i.e., primary control striving), they are likely to change their attitudes and create positive valuations (i.e., secondary control striving) that not only support the continued striving for primary control, but also control striving across domains (Heckhausen et al., 2010, 2019). In this sense, we propose that social participation is a personal resource that facilitates primary control striving across the lifespan, and through doing so people engage in secondary control strategies (attitudinal changes) which benefits not only their continued social participation, but also their life satisfaction and control capacity at work (i.e., their work ability).

### Social Participation as an Antecedent of Work Ability and Life Satisfaction

Social participation has been linked to improvements in cognitive sharpness, overall health and subsequent longevity, in part through an increase in hippocampal volume (Carlson, 2021). In fact, it was revealed that cognitive abilities of older adults involve considerable neuroplasticity and that skill maintenance and development serves as a predictor of cognitive functioning (Hultsch, et al., 1999; Carlson, 2021). Therefore, it can be assumed that continued skill development through social engagement can increase important work and life outcomes such as work ability and life satisfaction.

#### Work Ability and Life Satisfaction

Work ability is understood as one’s perception of their ability to meet the demands of their job and tends to lower with age as cognitive functioning and overall health declines (Cadiz et al., 2018; Hultsch et al., 1999; Ryff & Singer, 1998). Life satisfaction, on the other hand, has been noted as a judgement of one’s wellbeing and can vary as a function of one’s productivity and the quality of their relationships—both of which are predicted by social participation (Lachman et al., 2015). Interestingly, engagement-oriented individuals tend to experience more life satisfaction than pleasure-oriented individuals (Park et al., 2009). Further, these results were revealed crossculturally (Park et al., 2009), thereby suggesting that social participation is a global predictor of life satisfaction and overall wellbeing.

In line with the JD-R which posits that an individual’s work ability can vary as a function of the resources available to buffer the stress of their job’s demands, it has been established that a variety of psychosocial factors influence one’s work ability (Brady et al., 2019). Considering the JD-R’s emphasis on personal resources, such as health and mindset, we propose that social participation, which improves goal achievement in general (Carlson, 2021), has positive effects on work ability and life satisfaction

**Hypothesis 1**. Social participation is positively associated with work ability and life satisfaction.

### The Impact of Social Participation on Work Ability and Life Satisfaction: Sense of Control, Purpose in Life and Optimism as Mediators

Social participation has been associated with neurocognitive health through a variety of pathways (Carlson, 2021). For example, physical activity, whether for fitness or social events, improves sleep (Wang & Boros, 2021). Given that social participation contributes to improved psychosocial health, we propose that a person should have better psychosocial functioning in the form of: (a) increased optimism since ones basic psychological need for relationships is satisfied (Deci et al., 2017), (b) a greater sense of direction and purpose in life as one feels part of a community, and (c) a sense that their (voluntary) participation on social activities allows them to feel in control, satisfying one’s basic psychological need for autonomy (Deci et al., 2017). Social participation, such as through volunteering, can signal goal achievement (Jiang et al., 2018), which should also serve as facilitators of sense of control, purpose in life and optimism. Further, social participation encourages enthusiasm, passion and a sense of connection to functions larger than oneself (Park et al., 2009). Therefore, it can be expected that social participation will again be linked to sense of control, purpose in life, and optimism—in large part due to satisfying people’s basic psychological needs for autonomy and relatedness (Deci et al., 2017). In what follows, we elaborate on the relevance of self-determination theory (SDT; Deci & Ryan, 2008; Deci et al., 2017) for our model of social participation, as well as the biological underpinnings of our proposed model.

#### Sense of Control

According to self-determination theory (SDT; Deci et al., 2017), individuals share a core need to feel autonomous and, thus, that they can regulate their own behavior through free will (Sheldon, et al., 2020). Autonomy, which can be likened to a sense of control and has been noted as a valuable personal resource and linked to improvements in family, work and leisure domains while also serving as a predictor of achievement and health (Brady et al., 2019; Koestner & Holding, 2021). In fact, a higher sense of control has been associated with anti-inflammatory properties and a reduction of stress-inducing cortisol (Hong, et al., 2021). As a result, individuals with a heightened sense of control experience better longevity of life, perhaps through the pathway of emotional regulation and healthpromoting behaviors, which have been tied to work ability and life satisfaction (Lachman, et al., 2015; Cadiz, et al., 2018; Hong, et al., 2021).

**Hypothesis 2a.** Sense of control will mediate the relationship between social participation and work ability.
**Hypothesis 2b.** Sense of control will mediate the relationship between social participation and life satisfaction.

#### Purpose in Life

Purpose in life can be conceptualized as the base of a fulfilling life in the contexts of satisfaction, self-esteem and physical health (Park, et al., 2009). Interestingly, purpose in life emerged as a predictor of lower cortisol and inflammation as well as higher use of preventative health care, thereby offering protection from age-related biological declines while simultaneously promoting continued development and cognitive engagement (Carlson, 2021; Kim, et al., 2014). Thus, the attribution of meaning to one’s existence implies a variety of tangible benefits that can, in turn, predict work ability and life satisfaction.

**Hypothesis 3a.** Purpose in life will mediate the relationship between social participation and work ability.
**Hypothesis 3b.** Purpose in life will mediate the relationship between social participation and life satisfaction.

#### Optimism

**Figure 1:**
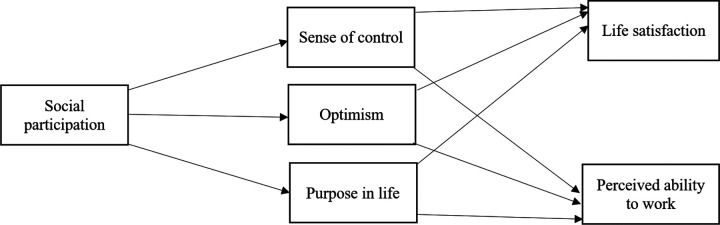
Conceptual model of study variables

Positive emotions signify that an achieved outcome is desirable and increase satisfaction of life, which then spills over to work outcomes (Diener & Seligman, 2004). Optimism can be characterized as a life orientation and has been noted as a particularly important personal resource in the context of the JDR (Brady et al., 2019). It has been found that optimism increases productivity, wellbeing and physical health through the buffering of stress and anxiety, boost to the immune system and promotion of connection to others (Diener & Seligman, 2004). Importantly, through social participation, a person should be optimistic because they are satisfying their innate need for relationships (Deci et al., 2017). Thus, considering the benefits of positive emotions and life orientations, as well as feeling part of a larger community, it seems feasible that optimism should serve as a predictor of both work ability and life satisfaction. See Figure 1 for a conceptual model of study variables.

**Hypothesis 4a.** Optimism will mediate the relationship between social participation and work ability.
**Hypothesis 4b.** Optimism will mediate the relationship between social participation and life satisfaction.

### The Present Study

Consistent with the JD-R, it is proposed that social participation is a personal resource that enhances life satisfaction and improves work ability in older workers. Integrating the MTLD with the JD-R, it is suggested that social participation represents primary control striving, while also fostering positive attitudinal changes through selective secondary control. Specifically, engaging in social participation promotes a greater sense of control, purpose in life, and optimism. These benefits not only enhance life satisfaction but also serve as additional resources, thereby helping individuals meet job demands more effectively.

The present study used 2018 and 2020 data from the Health and Retirement Study (HRS) for several reasons. First, the HRS provides a nationally representative longitudinal sample of older adults, enhancing the generalizability of our findings. Two waves to were used to evaluate the replication of results, thereby ensuring robustness. Second, the sample of workers aged 50+ is particularly vulnerable to declines in personal resources and health, making social participation crucial for maintaining mental health and work ability, especially as individuals approach retirement. To this end, using more recent HRS data allows the current study to provide timely insights to both scholars and practitioners. Finally, the study is among the few HRS-related papers that apply a process model focused on the importance of social participation in older workers (*cf* Beier et al., 2020), highlighting an underexplored area in research on occupational health and wellbeing.

#### Funding declaration

No funding was obtained for the current study.

## Method

### Participants

The current study draws from the 2018 and 2020 waves of the national Health and Retirement Study (HRS), which was designed to capture a variety of longitudinal phenomena in the realm of health, retirement and aging. Informed consent was obtained from each participant in the HRS. Specifically, in the 2018 wave, participants include 1,933 working individuals with an average age of 61.8 years (*SD* = 7.2). Further, participants reported an average of 13.58 years of education (*SD* = 3.0). In the 2020 wave, participants include an entirely different set of 1,427 working individuals with an average age of 62.2 years (*SD* = 7.3). Further, participants reported an average of 13.64 years of education (*SD* = 3.02). See Tables 1 and 2 for study demographics. There was no overlap between participants in each wave. Importantly, examining the study hypotheses with large samples improves statistical power, reduces sampling error and, ultimately, achieves a higher level of precision within results (Baker et al., 2021).

### Measures

#### Social Participation

Social participation was captured with 20 items that cover the frequency of participation in a variety of social activities (2018 *α* = .75, 2020, *α* = .75). For example, participants were asked to indicate how often they, “Do volunteer work” and “Go to a sport, social or other club.” All items were recorded on a 7-point Likert scale ranging from 1 (*daily*) to 7 (*never*). Importantly, items were reverse coded prior to statistical analysis to enhance clarity of the results.

#### Sense of Control

Perceived mastery items were employed to assess levels of personal sense of control (2018 *α* = .90, 2020 *α* = .91). Participants were asked to indicate their degree of agreement with five items including, “I can do just about anything I set my mind to” on a 6-point Likert scale ranging from 1 (*strongly disagree*) to 6 (*strongly agree*).

#### Purpose in Life

Purpose in life was measured using a subscale of the 1989 Ryff Measure of Psychological Wellbeing (2018 *α* = .76, 2020 *α* = .77). In particular, participants were asked to respond to seven items on a 6-point Likert scale ranging from 1 (*strongly disagree*) to 6 (*strongly agree*). Items include, “I enjoy making plans for the future and working to make them a reality.”

#### Optimism

Optimism items from the Life-Orientation Test – Revised (LOT-R) were employed (2018 *α* = .82, 2020 *α* = .82). Specifically, participants indicated their agreement with three items such as, “I’m always optimistic about the future,” on a 6-point Likert scale ranging from 1 (*strongly disagree*) to 6 (*strongly agree*).

#### Work Ability

The perceived ability to work scale incorporated four items to tap into participants’ ability to meet their job’s demands (2018 *α* = .95, 2020 *α* = .95). For example, they were asked to think about the “physical,” “mental” and “interpersonal” demands of their job and indicate the degree to which they were currently able to meet those demands on a 11-point Likert scale ranging from 0 (*cannot currently work at all*) to 10 (*work ability at its lifetime best*).

#### Life Satisfaction

Life satisfaction was measured with Diener’s measure of self-evaluated life quality (2018 *α* = .89, 2020 *α* = .88). Participants were instructed to respond to five items on a 7-point Likert scale ranging from 1 (*strongly disagree*) to 7 (*strongly agree*). Items include, “In most ways, my life is close to ideal.”

#### Controls

We controlled for age, gender and education to test for confounding effects and assure that our variables of interest served as unique predictors of the outcomes.

#### Data availability declaration

Data are publicly available on the HRS website.

### Analyses

The current study employed SPSS (version 29.0.2) PROCESS which uses 1,000 bootstrapped estimation (Hayes, 2013), to test for mediation. Specifically, sense of control, purpose in life and optimism were assessed as mediators of the relationship between social participation and both work ability and life satisfaction.

## Results

**Figure 2:**
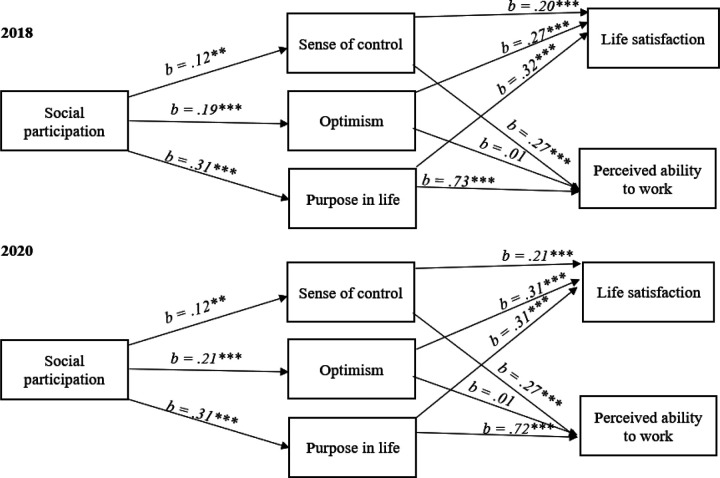
Mediators of the relationships between social participation and perceived ability to work and life satisfaction in 2018 (top) and 2020 (bottom) * p < .05; ** p < .01; *** p <.001

See Tables 3 and 4 for 2018 and 2020 correlations of all study variables, Tables 5 and 6 for regression statistics and Figure 2 for mediation statistics.

### Social participation, Work Ability and Life satisfaction

As expected, social participation was positively linked to both work ability and life satisfaction, thereby supporting Hypothesis 1. Importantly, results were consistent across waves. Specifically, in 2018 and 2020, social participation had a direct effect on work ability (*b* = .17 (2018), *b* = .17 (2020)) and life satisfaction (*b* = .13 (2018), *b* = .12 (2020)).

#### Sense of Control as a Mediator

In both HRS cohorts (2018 and 2020), sense of control was significantly positively correlated with each of the other study variables. Results drawn from 2018 data revealed that sense of control is a significant mediator of the relationship between social participation and work ability, 95% CI [.01, .06]. Moreover, these results were consistent in 2020, 95% CI [.01, .07], thereby offering support for Hypothesis 2a. In support for Hypothesis 3a, sense of control was also a significant mediator of the relationship between social participation and life satisfaction in both 2018, 95% CI [.06, .12], and 2020, 95% CI [.14, .26].

#### Purpose in Life as a Mediator

In both HRS cohorts (2018 and 2020), purpose in life was significantly positively correlated with each of the other study variables. Analyses from the 2018 wave indicate that purpose in life is a significant mediator of the relationship between social participation and work ability, 95% CI [.17, .30]. Data from the 2020 waves are consistent with this trend, 95% CI [.17, .22]. In addition, purpose in life also emerged as a significant mediator of the relationship between social participation and life satisfaction in both 2018, 95% CI [.07, .13], and 2020, 95% CI [.08, .12]. Hence, Hypotheses 2b and 3b were also supported.

#### Optimism as a Mediator

Finally, across both HRS cohorts (2018 and 2020), optimism was significantly positively correlated with each of the other study variables. Surprisingly, optimism was not a significant mediator of the relationship between social participation and work ability in 2018, 95% CI [−.02, .03], or 2020, 95% CI [−.01, .02]. On the other hand, 2018 results suggested that optimism is a significant mediator of the relationship between social participation and life satisfaction, 95% CI [.02, .09]. These results were consistent in the analyses of the 2020 wave, 95% CI [.08, .12]. Thus, whereas Hypothesis 2c was not supported, Hypothesis 3c was supported.

## Discussion

### Theoretical Implications

The current research builds on the existing literature by offering a pathway for the impact of social participation on work ability and life satisfaction. Previous research has noted the benefits of social participation (Wang & Boros, 2021; Jiang et al., 2020; Cadiz et al., 2018). Aligning with and expanding on this line of work the mechanisms by with these relationships function have been identified by the current research. Specifically, it was revealed that social participation promotes a sense of control, purpose in life and optimism. Moreover, whereas sense of control and purpose in life can predict both work ability and life satisfaction, optimism can predict life satisfaction. Hence, as expected, direct effects were uncovered. In addition, indirect effects were found. In particular, sense of control and purpose in life mediated the relationship between social participation and work ability as well as the relationship between social participation and life satisfaction. Finally, optimism mediated the relationship between social participation and life satisfaction. Thus, the current research fills a gap in the literature by revealing specific mechanisms driving the benefits of social participation. The observed results were replicated between 2018 and 2020 waves of HRS data, thereby offering a unique strength of the current study and providing a stronger weight of evidence.

Importantly, the current research can serve to inform future work. For example, using the current findings as a base, future research may seek to investigate the growing importance and relevance of social participation as a personal resource and, perhaps, expand the personal resource dimension of the JD-R to include social participation and related concepts. Moreover, the current research may guide future work to study the noted relationships on a longitudinal level, which may offer insight regarding long-term health outcomes, career sustainability and organizational growth.

### Practical Implications

In addition to theoretical contributions, the current research offers practical insights and has the potential to positively influence both employees and their organizations. It has been noted that, as individuals age, they experience a shift in orientation towards emotional goals such as contributing to society and staying close with family (Jiang et al., 2020). In this context, it is likely that social participation can signify emotional goal achievement, thereby boosting sense of control, purpose in life, and optimism and subsequent positive outcomes. Even so, younger populations should be encouraged to actively engage with their social environments as a form of preventative medicine and predictor of long-term health and wellness. Particularly, building sustainable habits for mental and physical health can improve coping, reduce risk of chronic disease and, ultimately, optimize lifelong wellbeing (Kim et al., 2014). Therefore, the current research can be used as a foundation to guide individuals toward cognitive, social, and physical longevity.

While the research poses clear benefits to individuals, it also serves to aid organizations. Life satisfaction and work ability have been linked to productivity (Brady et al., 2020). Thus, life satisfaction and work ability among employees may buffer absenteeism and improve organizational output, thereby minimizing unnecessary costs and optimizing growth.

### Limitations and Future Research

The present study has limitations. First, there are inherent limitations with the use of cross-sectional data in the context of mediation. Since data in both waves were collected at single timeframes, temporal precedence cannot be determined. Thus, the presented model offers only one of the possible directionalities between the explored variables and causality cannot be determined. Even so, the study does provide a meaningful initial examination of potential mediating pathways that can serve as a sound foundation for future work. Additionally, even though the cross-section results were consistent over time, the study was not longitudinal. Thus, it would be interesting to investigate the impact of long-term social participation, perhaps throughout the duration of one’s career. Similarly, the sample was limited to older workers (primarily aged 50 + years old). Future work may include a younger sample to determine how social participation can be used as a long-term form of preventative medicine. Finally, the HRS social participation measure was broad with social activities ranging from spending time with one’s grandchildren to engaging in religious rituals. Researchers should consider investigating more specific means of social participation, such as volunteering, group fitness and art clubs, to uncover the most efficient strategies of boosting sense of control, purpose in life and optimism and subsequent outcomes.

## Conclusions

Considering the trend of an aging workforce, the notion of poor health in the U.S. and the prediction that the working population of older adults will double by 2050, the literature has called for preventative medicine (Kim et al., 2014), perhaps in the form of lifestyle features, such as social participation. The current study found that sense of control and purpose in life mediated the positive relationship between social participation and work ability. Further, sense of control, purpose in life, and optimism mediated the positive relationship between social participation and life satisfaction. Importantly, all results were consistent across the 2018 and 2020 HRS cohorts, which represents aging workers from a variety of socioeconomic and educational backgrounds across the United States. These findings contribute to the aging, wellbeing and occupational health literature while also posing a variety of practical implications.

## Figures and Tables

**Table 1:** Study demographics (2018) n = 1933

Characteristic	Percentage
**Age (years, range 50–90)**	
50–60	52
61–70	35
71–80	10
81+	3
**Gender**	
Male	44
Female	56
**Education (years, range 0–17)**	
0–4	2
5–9	6
10–15	56
16+	36

**Table2:** Study demographics (2020) n = 1,427

Characteristic	Percentage
**Age (years, range 50–94)**	
50–60	46
61–70	42
71–80	8
81+	4
**Gender**	
Male	43
Female	57
**Education (years, range 0–17)**	
0–4	2
5–9	7
10–15	54
16+	37

**Table 3:** Correlation Matrix of Study Variables (2018)

Variable	1	2	3	4	5	6	7	8	9
1. Social participation	-								
2. Sense of control	.08**	-							
3. Purpose in life	.26***	.34***	-						
4. Optimism	.13***	.44***	.42***	-					
5. Work ability	.20***	.25***	.36***	.19***	-				
6. Life satisfaction	.13***	.33***	.35***	.37***	.17***	-			
7. Age	.07*	−.02	−.03	.01	−.23**	.07*	-	-	-
8. Education	−.10**	.07**	.20**	.09*	.32**	.05*	−.01*	−.07*	
9. Gender	−.16*	−.01	.02	−.01	−.02	−.03	−.03		

** p <.01;

*** p <.001

**Table 4:** Correlation Matrix of Study Variables (2020)

Variable	1	2	3	4	5	6	7	8	9
1. Social participation	-								
2. Sense of control	.14**	-							
3. Purpose in life	.27***	.39***	-						
4. Optimism	.19***	.40***	.36***	-					
5. Work ability	.19***	.30***	.30***	.19***	-				
6. Life satisfaction	.17***	.31***	.33***	.38***	.27***	-			
7. Age	.06*	−.03	−.03*	.01	−.27**	.05*	-	-	-
8. Education	.29**	.07*	.19**	.09*	.31**	.07*	.05*	−.02	
9. Gender	−.17*	−.02	.02	−.01	−.03	−.02	−.01		

** p <.01;

*** p <.001

**Table 5:** Regression Results (2018)

Model	Parameter (β)
a. Life satisfaction predicted by:	
Social participation	0.13**
Purpose in life	0.32***
Optimism	0.28***
Sense of control	0.21***
Age	0.03**
Gender	−0.01
Years of education	−0.02
b. Work ability predicted by:	
Social participation	0.15**
Purpose in life	0.61***
Optimism	0.01
Sense of control	0.24***
Age	−0.08***
Gender	−0.28**
Years of education	0.17***

* p<.05;

** p<.01;

*** p<.001

parameters are standardized beta coefficients (β)

**Table 6:** Regression Results (2020)

Model	Parameter (β)	SE
a. Life satisfaction predicted by:		
Social participation	0.19**	0.029
Purpose in life	0.34***	0.033
Optimism	0.31***	0.018
Sense of control	0.22***	0.019
Age	0.02***	0.002
Gender	−0.14*	0.060
Years of education	−0.01	0.011
b. Work ability predicted by:		
Social participation	0.17*	0.079
Purpose in life	0.62***	0.093
Optimism	0.09	0.052
Sense of control	0.21***	0.056
Age	−0.05***	0.007
Gender	−0.19	0.102
Years of education	0.18***	0.019

* p<.05;

** p<.01;

*** p<.001

parameters are standardized beta coefficients (β)

## Data Availability

Data are publicly available on the HRS website: https://hrsdata.isr.umich.edu/data-products/public-survey-data?_gl=1*1mxjd4c*_ga*MTAxNzY3NDg3Ni4xNzI2NjA1NTc0*_ga_FF28MW3MW2*MTcyNjYwNTU3NC4xLjAuMTcyNjYwNTU3NC4wLjAuMA. The link is embedded within the text in the manuscript.
